# H2G-Net: A multi-resolution refinement approach for segmentation of breast cancer region in gigapixel histopathological images

**DOI:** 10.3389/fmed.2022.971873

**Published:** 2022-09-14

**Authors:** André Pedersen, Erik Smistad, Tor V. Rise, Vibeke G. Dale, Henrik S. Pettersen, Tor-Arne S. Nordmo, David Bouget, Ingerid Reinertsen, Marit Valla

**Affiliations:** ^1^Department of Clinical and Molecular Medicine, Norwegian University of Science and Technology, Trondheim, Norway; ^2^Clinic of Surgery, St. Olavs Hospital, Trondheim University Hospital, Trondheim, Norway; ^3^Department of Health Research, SINTEF Digital, Trondheim, Norway; ^4^Department of Circulation and Medical Imaging, Norwegian University of Science and Technology, Trondheim, Norway; ^5^Department of Pathology, St. Olavs hospital, Trondheim University Hospital, Trondheim, Norway; ^6^Department of Computer Science, UiT The Arctic University of Norway, Tromsø, Norway; ^7^Clinic of Laboratory Medicine, St. Olavs hospital, Trondheim University Hospital, Trondheim, Norway

**Keywords:** hybrid guiding, refinement network, deep learning, convolutional neural networks, digital pathology, hierarchical sampling, clustering, breast cancer

## Abstract

Over the past decades, histopathological cancer diagnostics has become more complex, and the increasing number of biopsies is a challenge for most pathology laboratories. Thus, development of automatic methods for evaluation of histopathological cancer sections would be of value. In this study, we used 624 whole slide images (WSIs) of breast cancer from a Norwegian cohort. We propose a cascaded convolutional neural network design, called H2G-Net, for segmentation of breast cancer region from gigapixel histopathological images. The design involves a detection stage using a patch-wise method, and a refinement stage using a convolutional autoencoder. To validate the design, we conducted an ablation study to assess the impact of selected components in the pipeline on tumor segmentation. Guiding segmentation, using hierarchical sampling and deep heatmap refinement, proved to be beneficial when segmenting the histopathological images. We found a significant improvement when using a refinement network for post-processing the generated tumor segmentation heatmaps. The overall best design achieved a Dice similarity coefficient of 0.933±0.069 on an independent test set of 90 WSIs. The design outperformed single-resolution approaches, such as cluster-guided, patch-wise high-resolution classification using MobileNetV2 (0.872±0.092) and a low-resolution U-Net (0.874±0.128). In addition, the design performed consistently on WSIs across all histological grades and segmentation on a representative × 400 WSI took ~ 58 s, using only the central processing unit. The findings demonstrate the potential of utilizing a refinement network to improve patch-wise predictions. The solution is efficient and does not require overlapping patch inference or ensembling. Furthermore, we showed that deep neural networks can be trained using a random sampling scheme that balances on multiple different labels simultaneously, without the need of storing patches on disk. Future work should involve more efficient patch generation and sampling, as well as improved clustering.

## 1. Introduction

Cancer is an important cause of death, and of all cancers, breast cancer has the highest incidence worldwide (1). Cancer diagnostics is based on clinical examination, medical imaging and histopathological assessment of the tumor. The latter includes analysis of specific biomarkers that often guides treatment of the patients. Most pathology laboratories are burdened by an increasing number of biopsies and more complex diagnostics (2). To reduce workload for pathologists, automatic assessment of tumors and biomarkers would be of value.

A natural first step in automatic tumor and biomarker analysis would be to correctly identify the lesion, thus separating the tumor from surrounding tissue. For automatic biomarker assessment, it is important to ensure that biomarker status is obtained exclusively in the invasive epithelial cancer cells. With the promise of deep learning-based methods in computational pathology (3), accurate segmentation of the cancer region would be beneficial for building new classifiers and facilitate other cancer analysis methods.

Processing histopathological WSIs is challenging due to their large size. WSIs captured at × 400 magnification may be as large as 200*k*× 100*k* pixels, and as such, cannot be used directly as input to convolutional neural networks (CNNs). A solution is to downsample the image to a size that is manageable for the CNN. However, this results in loss of information and is therefore often not useful for tumor segmentation. Another widely used approach is to divide the image into smaller patches (4), before each patch is sent to an algorithm to produce an output. The results are then stitched to form a complete segmentation or heatmap of the entire WSI. However, the use of such a patch-wise design based on high-resolution information only, often results in edge artifacts and poor global segmentation of larger structures (5).

Traditional segmentation methods such as geodesic active contour (6) and superpixel (7) have been used for segmentation of histopathological images. In recent years, deep learning-based methods have surpassed traditional methods. From the challenge paper of CAMELYON16 (8), all 32 participating teams used machine learning-based methods, of which 25 teams used deep learning-based methods. The seven non-deep learning-based methods performed poorest on both the semantic segmentation and classification tasks. We therefore only consider deep learning-based approaches in this study.

Schmitz et al. (9) compared multi-scale convolutional autoencoder (CAE) designs, applied in a patch-wise fashion across liver tumors in WSIs. They found that the network benefited significantly from the added multi-scale information, compared to the baseline U-Net (10). They also proposed non-overlapping inference to reduce runtime at the cost of reduced accuracy along patch edges. For handling these edge artifacts, Priego Torres et al. (5) proposed a conditional random field-based, patch-wise, merging scheme.

To improve the patch-wise design, Guo et al. (11) developed a multi-task network for classification and semantic segmentation of breast cancer. They used a pretrained InceptionV3 (12) architecture and fine-tuned it on the CAMELYON16 data set (8). Such transfer learning has the benefit of making training more efficient, as the network is not trained from scratch. Using a more complex backbone, such as InceptionV3, has the potential benefit of improved performance. However, the architecture is computationally expensive, and might therefore not be suitable for real-time applications, such as histopathological diagnostics.

Breast cancers are known for their intra- and intertumor heterogeneity, and thus their morphological appearance varies both within and between tumors. Due to intratumor heterogeneity, the patches generated from a single WSI often contain different tissue types and a varying morphological appearance. Qaiser et al. (13) studied the effect of smart patch selection and balancing in preprocessing, to produce models that performed well on varying types of tissue. They demonstrated that a deep clustering approach of patches outperformed the conventional *k*-means (14) clustering method.

A similar cluster-guiding strategy was performed by (15) using multiple instance learning. They used a pretrained VGG19-encoder (16) for feature extraction. The dimensionality of the features was reduced using principal component analysis (PCA) (17), before performing *k*-means clustering. Samples were drawn from these clusters and balanced during training. The number of clusters was set to four, as they assumed that there were four main natural tissue types in the data.

For image classification, MobileNetV2 (18) and InceptionV3 (12) are popular baseline architectures, commonly used in digital pathology (4, 19, 20). For image segmentation, the most commonly used CAE is U-Net (10). To make the U-Net design more efficient, various concepts have been proposed, such as multi-scale input (21), deep supervision (22), and attention (23). These three concepts are used in the two segmentation architectures AGU-Net and DAGU-Net (24). Performing both detection and semantic segmentation in a single step is a challenge. The segmentation result is often suboptimal, and therefore a post-processing method is required. Refinement networks for the CAE itself have therefore been proposed, either in multiple steps (25) or end-to-end, e.g., DoubleU-Net (26).

We propose a novel cascaded CNN design, H2G-Net, which efficiently utilizes both high-resolution and global information. The design is rapid, does not require ensembling, and can be used on low-end hardware. H2G-Net is a two-stage design. In the first stage, initial segmentation of the tumor region is extracted by applying a patch-wise classifier model. To correct for the fragmentation and pixelation in the generated tumor heatmap, a refinement network is used in the second stage. The network utilizes both the tumor heatmap and a low-resolution version of the WSI to produce the final segmentation. To counter the heterogeneity of breast cancer, a novel sampling scheme is proposed, which simplifies training by handling multiple data imbalance challenges simultaneously and reads patches directly from the raw WSI format during training.

In this paper, we present the following contributions:

(1) A new, large data set of 624 breast cancer WSIs annotated by pathologists.
(2) A novel sampling scheme that extracts patches directly from the WSI, handles hierarchical structured data imbalance problems, and enables end-to-end cluster-guiding.
(3) A novel approach where a cascaded CNN combines high-resolution and global information in histopathological images, producing superior performance over single-resolution approaches.
(4) The proposed pipeline and trained models are made openly available for use in FastPathology (27).

## 2. Materials and methods

### 2.1. Data set and annotation design

In this study, we used 4 μm thick whole sections (*n* = 624) from a cohort of Norwegian breast cancer patients (28), Breast Cancer Subtypes 1 (BCS-1). All tumors were previously classified into histological grade, according to the Nottingham grading system (29). The sections were stained with hematoxylin-eosin (H&E), scanned at × 400 magnification using an Olympus scanner BX61VS with VSI120-S5, and stored in the cellSens VSI format using JPEG2000 compression.

For each WSI, the tumor area was delineated by pathologists using QuPath (30). The tumor area included not only invasive epithelial cells, but also surrounding stromal tissue including other cells such as fibroblasts and inflammatory cells, and blood vessels. It is well-known that these components are also important for cancer development (31–33), and thus including these in the annotation could be of value in future studies of cancer progression and prognostication. To speed up and assist with the annotation work, automatic and semi-automatic approaches were tested, similarly to the approach used by (34). We used two different approaches for annotation (AN1 and AN2). For both annotation designs, predicted annotations were manually adjusted by the pathologists using the brush tool in QuPath.

The first 150 WSIs were annotated using the **AN1** method, which involved using the semi-automatic tissue detection function in QuPath. The following parameters were used for performing segmentation: simple tissue detection threshold 200, requested pixel size 20, and minimum area 100,000. In cases were the algorithm failed, the parameters were adjusted or the tumor was manually annotated from scratch.

**Figure 1:**
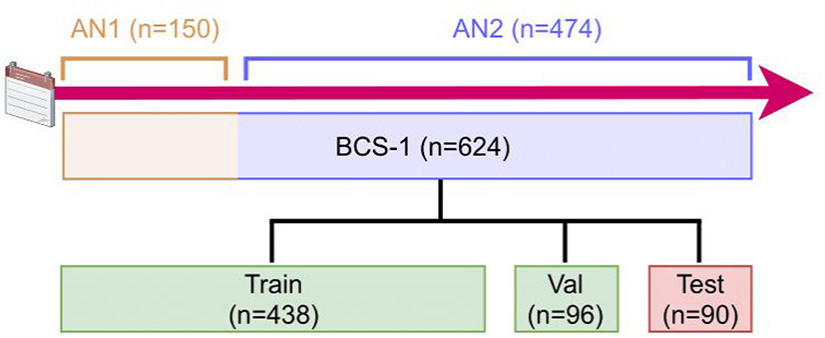
Description of the data generation timeline and process. The 624 WSIs were annotated with two different annotation methods (AN1 and AN2). The data set was then randomly split into train, validation, and test sets. AN, Annotation; BCS, Breast Cancer Subtypes; Val, Validation.

The remaining WSIs (*n* = 474) were annotated using the **AN2** method (see Figure 1). A patch-wise CNN, similar to the Inc-PW method described in Section 3.2, was trained from a subset of the first 150 annotated WSIs. The model was trained in Python, and the produced model was then applied to the remaining WSIs. The resulting heatmaps were imported in QuPath and converted to annotations. Simple morphological post-processing was then performed before the segmentations were adjusted by the pathologists. Finally, to ensure consistency, all annotations were reviewed by a single pathologist experienced in breast cancer pathology and minor adjustments were made.

The pathologists' annotations were exported from QuPath as individual PNGs, one for each WSI, with a downsampling factor of four. The PNGs were then converted to tiled, pyramidal TIFFs, using the command line tool vips^1^, with tiles sized 1024 × 1024 and a LZW lossless compression. All WSIs were converted to the single-file, pyramidal tiled, generic TIFF format using the command line tool vsi2tif^2^. Lastly, the annotated WSIs were randomly distributed into the three sets: training (~70%; *n* = 438), validation (~15%; *n* = 96), and test (~15%; *n* = 90) set.

### 2.2. Preprocessing

The tissue regions of each WSI were automatically segmented by the following steps: (1) Extract the × 1.25 image plane of the WSI, (2) convert the image to the HSV (Hue, Saturation, Value) color domain, (3) extract and threshold the saturation channel image using a fixed threshold of 20, (4) perform two consecutive applications of morphological closing using kernel sizes 5 × 5 and 3 × 3, respectively.

Following annotation, we extracted patches sized 256 × 256 at × 100 magnification level from the WSIs. Only patches containing more than 25% tissue were included. Patches with more than 25% tumor were considered tumor patches, and only patches with no tumor were considered non-tumor. The remaining patches in the range (0, 25]% tumor tissue were discarded. For each WSI, the coordinates of accepted patches were stored along with the assigned label i.e., non-tumor/tumor.

### 2.3. Hierarchical sampling scheme

A batch generator was created to sample patches directly from the raw WSI format. Patches were read using OpenSlide (35), which enabled multi-threading processing. The generator was based on the condition that it is important to balance patches according to the following features: class label, tissue type, tissue and tumor area, and histological grade.

**Figure 2:**
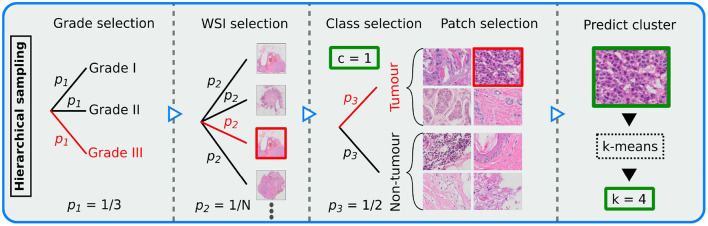
Illustration of the hierarchical sampling scheme, demonstrating how patches were sampled from the *N* whole slide images (WSIs) for training the patch-wise model. Sampling was conducted as a uniform tree diagram. Thus, *p*_*i*_ represents probability at step *i*∈{1, 2, 3}. A potential path for patch selection is marked red. Each patch was assigned a class label *c* (tumor or non-tumor) and a cluster *k* (10 different clusters). Each output is marked in green.

Patches were sampled in a hierarchical sampling scheme (see Figure 2), conducted as a tree structure uniformly distributed at each respective stage. The goal was to make all relevant outcomes equally probable. The sampling scheme was defined in the ordered stages: (1) Randomly select a histological grade, (2) from the grade select a WSI, (3) from the WSI select a class label, (4) from the class label select a patch.

To include patch-level tissue type label in the balancing scheme, we used our sampling generator to train a *k*-means clustering model, similar to (15). From a set of 100 batches of size 32, features were extracted using a VGG-16 (16) backbone pretrained on the ImageNet data set (36). The extracted features were then standardized using Z-score normalization, before PCA was performed. The number of principal components was chosen such that 95% of the variance of the data was explained. The *k*-means model was then trained using *k* = 10 number of clusters, as recommended in a related study (37). The clustering model was implemented using the Python library scikit-learn (38).

To utilize the trained clustering model in the patch-wise CNN, TensorFlow (39) equivalents of the standardization, PCA and *k*-means transform methods were implemented, which was defined as a TensorFlow graph. The scikit-learn trained weights were then loaded for each corresponding component. Finally, for training the CNN classifier, each patch was passed through two different graphs; (I) a frozen pipeline that performed clustering and (II) a learnable deep neural network that performed classification. The outputs from both models were then passed to the loss function.

The MobileNetV2 (18) architecture was used for the patch-wise CNN classification of breast cancer tumor tissue, as it is lightweight, efficient, and optimized for low-end processors and thus suitable for real-time deployment. To further reduce the number of parameters, we simplified the classifier head. The updated classifier contained a global average pooling layer, followed by a dense layer of 100 hidden neurons, dropout (40) with a 50% drop rate, ReLU activation function, batch normalization (41), and finally a dense layer with softmax activation function.

### 2.4. Cluster-guided loss function

To balance on tissue type and thus ensure similar model performance on all predicted clusters, we included the cluster-information in the loss computation. By doing so, we enabled end-to-end cluster-guiding and made the clustering design more scalable, as the cluster heatmap does not need to be generated in a separate step or stored on disk. For a given batch, we calculated the cross-entropy loss for each cluster independently, and then calculated the macro average across each cluster. We named this loss function cluster-weighted categorical cross-entropy (CWCE) loss. The loss can be mathematically described as:


(1) $${ℒ}_{\text{CWCE}}=-\frac{1}{K_{b}}\sum_{k=1}^{K_{b}}\sum_{c=1}^{C}\sum_{i=1}^{B}1(q_{i,k}=k)y_{i,c}log(p_{i,c})\%\text{CWCE}=-\frac{1}{K}\sum_{k=1}^{K}\sum_{c=1}^{C}\sum_{i=1}^{B}p_{i,k}y_{i,c}\text{log}(p_{i,c})$$

where *i*∈{1, …, *B*} represents sample *i* in a batch of size *B*, *k*∈{1, …, *K*_*b*_} cluster in a mini-batch *b* of size *B* of *K*_*b*_ represented clusters, *c*∈{1, …, *C*} class, *p* class prediction, *q* cluster prediction, and ground truth tumor class. Note that the number of clusters *K*_*b*_ may vary between mini-batches.

### 2.5. Heatmap generation and refinement stage

**Figure 3:**
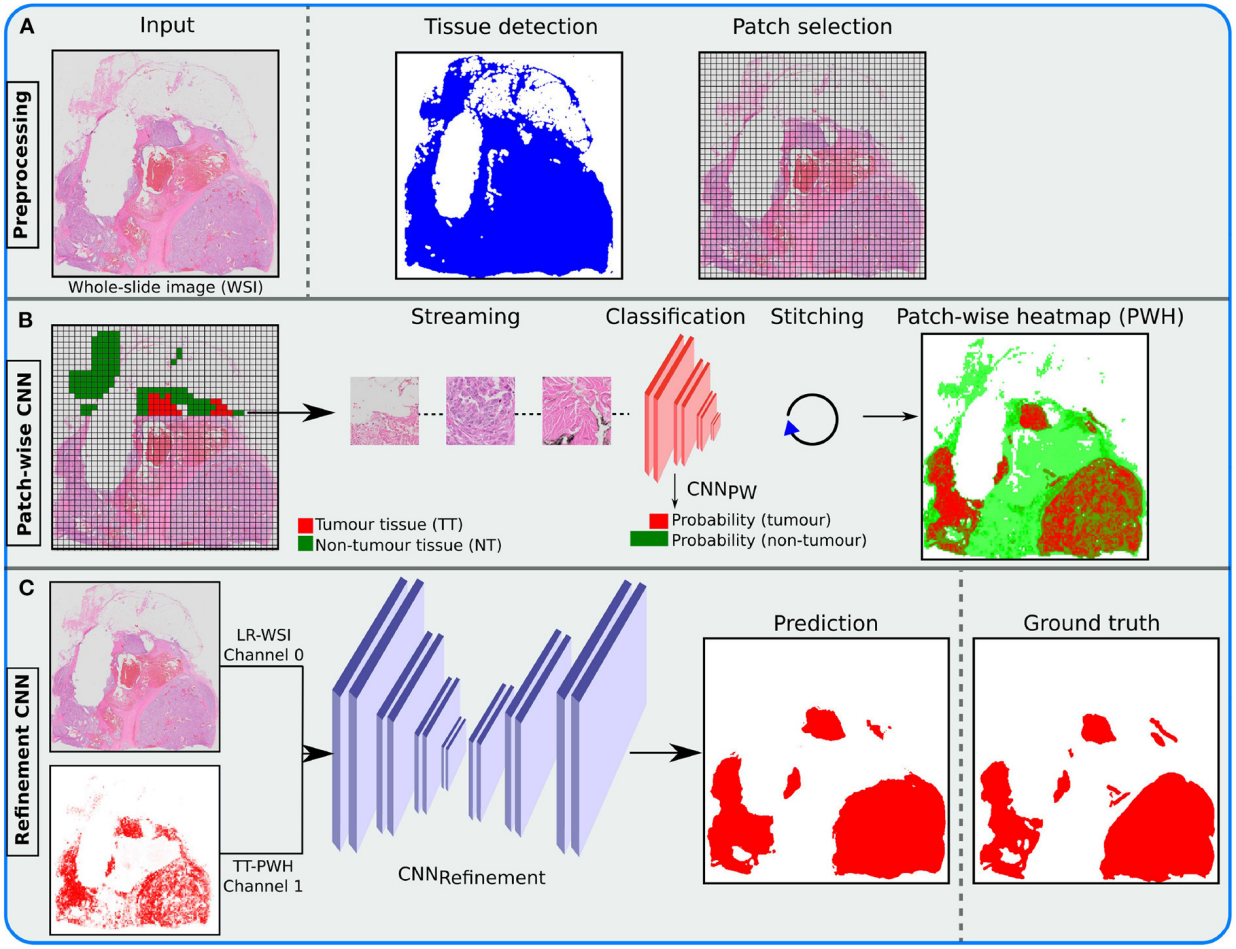
Illustration of the inference pipeline, from the whole slide image (WSI) to the final tumor segmentation (prediction). **(A)** Apply tissue detection before patch selection. **(B)** Stream accepted patches through a trained patch convolutional neural network (CNN) classifier and stitch the output to form a patch-wise heatmap (PWH). **(C)** Merge the low-resolution (LR) WSI with the resulting tumor tissue (TT) PWH and send it through the trained refinement CNN, using a probability threshold of 0.5, to produce the final prediction.

The tumor heatmaps were generated by: (1) extracting the tissue region as described in Section 2.2 and generating image patches from the tissue region (see Figure 3A), (2) applying the trained patch-wise model in a sliding window fashion across the WSI, and (3) and stitching the tumor class softmax prediction of each patch to form a tumor heatmap (see Figure 3B). Heatmaps were generated for all WSIs in the training and validation sets, as these were relevant for the second stage. Alternatively, if only a patch-wise model was used, these heatmaps were thresholded by 0.5 to produce segmentations of the tumor region. The latter represents the segmentation designs (III)-(V) mentioned in Section 3.2).

To improve the result from the patch-wise detection, we combined the heatmap with the low-resolution WSI (see Figure 3C). This was done in an additional stage using a Refinement CNN. A suitable magnification level was chosen (≥1024 × 1024 pixels), and a low-resolution version of the original WSI was extracted from the image pyramid. The image was then normalized to [0, 1], before both the resulting image and the heatmap were resized to 1024 × 1024 using bilinear interpolation. A separate fully-convolutional neural network was then used to refine the resulting heatmaps from the detection stage. We used the U-Net (10) architecture, which took the concatenated low-resolution three-channel WSI and predicted heatmap as input.

### 2.6. From development to deployment

After training the patch-wise and refinement models, the models are ready to be used for inference. The inference pipeline is illustrated in Figure 3. The trained TensorFlow models were converted to the ONNX (42) standard format, to enable efficient inference on both graphics processing unit (GPU) and central processing unit (CPU) with different frameworks. The models were then integrated into the FastPathology (27) platform by writing a FAST (43) text pipeline, containing information about the inference pipeline and how the models should be handled (e.g., input shape, node names, and inference type). Thus, the proposed pipeline can be used through a graphical user interface (GUI) without programming. Binary release of FastPathology, trained models, test data, and source code can be accessed on GitHub^3^.

## 3. Validation study

### 3.1. Experiments

We conducted an ablation study to evaluate our design. The experiments conducted were:

(1) To assess the importance of architecture complexity in breast cancer tumor detection in WSIs, we compared CNN classifiers using the two backbone architectures InceptionV3 and MobileNetV2.
(2) To evaluate the cluster-guiding approach, we conducted experiments with and without *k*-means using the MobileNetV2 backbone.
(3) To assess the effect of post-processing on the predicted heatmap, we compared state-of-the-art CAEs against simple baseline methods.
(4) To evaluate the importance of having a GPU for inference, runtime measurements of the best performing method were performed with and without using the GPU, using TensorRT (44) and OpenVINO (45) for GPU and CPU inference, respectively.

### 3.2. Baseline segmentation methods

Using our pipeline, any architecture or component can be removed, added, or substituted. It is therefore valuable to assess the importance of each component in the pipeline. To evaluate the pipeline, we used existing, well-documented, state-of-the-art architectures. For patch-wise classification we used the MobileNetV2 (~2.39M params.) and InceptionV3 (~22.00M params.) backbones pretrained on the ImageNet data set, and used the same simplified classifier head for both architectures, as described in Section 2.3.

For image segmentation refinement, we compared the CAE architectures U-Net (~11.58M params.), AGU-Net (~7.68M params.), DAGU-Net (~9.99M params.), and DoubleU-Net (16.04M params.). In addition, we included a traditional, widely used tissue segmentation method (46), to serve as a minimal baseline measure. This method simply segments all tissue, and thus all tuned methods should outperform it. For this method, the image was resized to 1024 × 1024, before being converted to the HSV color domain. Then, the saturation image was thresholded using Otsu's method (47).

The autoencoders were slightly modified to work better for our use case and data set. To make comparison fair, all autoencoders had similar depth and filter arrangement. Our U-Net architecture consisted of nine encoder and decoder blocks, with the following filters (from top to bottom): {8, 16, 32, 64, 128, 128, 256, 256, 512}. The encoder block used the following operations (Convolution-BatchNormalization-ReLU) × 2-MaxPooling. The AGU-Net, DAGU-Net, and DoubleU-Net architectures used the same filter arrangement as U-Net, but based on eight levels without the last 512 filter due to memory constraints. Furthermore, DoubleU-Net used 42 atrous spatial pyramid pooling filters. All autoencoders were symmetric, trained from scratch, modified to support two inputs (image and heatmap), and used softmax activation function in the last layer. Implementations of all architectures used in this study are openly available in our GitHub repository^4^.

In summary, the following segmentation designs were compared:

(I) **Otsu**: Intensity-based thresholding for tissue segmentation.
(II) **UNet-LR**: Segmentation of low-resolution WSI using a U-Net architecture.
(III) **Inc-PW**: Patch-wise classification using an InceptionV3 architecture.
(IV) **Mob-PW**: Patch-wise classification using a MobileNetV2 architecture.
(V) **Mob-KM-PW**: Same as (IV), with *k*-means guiding.
(VI) **Mob-PW-UNet**: Same as (IV), with a U-Net refinement network, without *k*-means guiding.
(VII) **Mob-PW-AGUNet**: Same as (IV), with an AGU-Net refinement network.
(VIII) **Mob-PW-DAGUNet**: Same as (IV), with a DAGU-Net refinement network.
(IX) **Mob-PW-DoubleUNet**: Same as (IV), with a DoubleU-Net refinement network.

### 3.3. Statistical evaluation

All patch-wise and refinement models were trained using the same training set, and the best models were selected based on the performance on the validation set. The test set was used as a hold-out sample for an unbiased, final evaluation.

As our use case is on breast cancer segmentation, we only consider binary segmentation in this study. However, H2G-Net supports multiclass semantic segmentation. A threshold of 0.5 was used to distinguish between the tumor and non-tumor classes. Metrics were reported WSI-wise, and only on the test set. For each respective metric, macro average and standard deviation were reported. The specific metrics used to assess performance were pixel-wise recall, precision, and the Dice similarity coefficient (DSC). To further assess the robustness of the design, we also reported DSC for each histological grade. We performed multiple pairwise Tukey's range tests, comparing the DSC measures for all deep learning-based designs. The p-values were estimated for the test set (see Supplementary Table 1).

### 3.4. Training parameters

For training the classification models, we fine-tuned the respective pretrained backbones using the Adam optimizer (48) with an initial learning rate of 1e-4. For batch generation, 500 and 200 batches of size 64 for training and validation, respectively, were sampled randomly for each epoch. The models were trained for 100 epochs. Batches were generated in parallel using eight workers with a maximum queue size of 20. Models were trained using the following online data augmentation scheme of which all had a 50% chance of being used: random horizontal/vertical flip, 90° lossless rotations, HSV color augmentation with a random shift of range [−20, 20], and multiplicative brightness augmentation of range [0.8, 1.2].

All segmentation models were trained from scratch using the Adam optimizer with an initial learning rate of 1e-3. Accumulated gradients using a batch size of four with six accumulation steps were performed. For online data augmentation, simple horizontal/vertical flip, 90° rotations, random zoom of range [0.8, 1.2], and Macenko (49) stain augmentation^5^ using σ_1_ = σ_2_ = 0.1, with a chance of 50% of being used, were conducted. The models were trained for 1,000 epochs, or until the early stopping criterion with a patience of 100 epochs was achieved.

Implementation was done in Python 3.6, and CNN architectures were implemented in TensorFlow (v1.13.1). Experiments were performed using an Intel Xeon Silver 3110 CPU, with 32 cores and 2.10 GHz, and an NVIDIA Quadro P5000 dedicated GPU.

## 4. Results

For the test set, all deep learning-based methods outperformed the tissue segmentation method, Otsu, in terms of DSC (see Table 1). Comparing the patch-wise classifiers, Inc-PW and Mob-PW, no significant difference in DSC was found between the architectures (*p*≈0.9, see Supplementary Table 1). Adding cluster-guiding to Mob-PW, Mob-PW-KM, slightly reduced performance, however, not significantly (*p*≈0.9). Among the best single-resolution designs (i.e., UNet-LR, Inc-PW, and Mob-PW), the patch-wise approaches performed slightly better in terms of DSC, but not significantly (*p*≈ 0.9). The low-resolution approach (UNet-LR) achieved better recall, but with the cost of poorer precision.

**Table 1:** Test set segmentation performance for the different designs.

	**Designs**	**Recall**	**Precision**	**Dice similarity coefficient**
(I)	Otsu	0.990 ± 0.027	0.534 ± 0.200	0.669 ± 0.179
(II)	UNet-LR	0.931 ± 0.113	0.851 ± 0.165	0.874 ± 0.128
(III)	Inc-PW	0.881 ± 0.118	0.909 ± 0.099	0.887 ± 0.089
(IV)	Mob-PW	0.879 ± 0.123	0.907 ± 0.100	0.885 ± 0.094
(V)	Mob-KM-PW	0.853 ± 0.124	0.909 ± 0.097	0.872 ± 0.092
(VI)	Mob-PW-UNet	0.944 ± 0.074	**0.929 ± 0.088**	**0.933 ± 0.069**
(VII)	Mob-PW-AGUNet	**0.954 ± 0.066**	0.909 ± 0.097	0.927 ± 0.072
(VIII)	Mob-PW-DAGUNet	0.942 ± 0.075	0.922 ± 0.091	0.928 ± 0.072
(IX)	Mob-PW-DoubleUNet	0.949 ± 0.073	0.919 ± 0.093	0.929 ± 0.074

DSC, Dice Similarity Coefficient; Inc, InceptionV3; Mob, MobileNetV2; PW, Patch-wise; KM, *k*-means; LR, Low-resolution. Results are reported as mean ± standard deviation.

Introducing a U-Net-inspired refinement network (using both the low-resolution WSI and the resulting heatmap from Mob-PW) resulted in significant improvement compared to the best single-resolution approach (Mob-PW-UNet vs. Inc-PW, *p*≈0.012). All methods using a refinement network significantly outperformed the single-resolution approaches. Comparing the refinement architectures, the best performance in terms of precision and DSC was found from the U-Net design, Mob-PW-UNet, but the difference was not statistically significant (*p*≈0.9 for all comparisons). No benefit of using more advanced CAE architectures was found.

When each histological grade was analyzed separately, Mob-PW-DoubleUNet was the best performing method for grade I and Mob-PW-UNet performed best on grade II and III (see Table 2). All designs guided by the hierarchical sampling scheme (designs (II)-(IX)) performed similarly across all histological grades, indicating that performance was somewhat invariant to histological grade.

**Table 2:** Test set segmentation performance for the different designs in histological grades I-III.

	**Designs**	**Dice similarity coefficient (*****n*** **= 90)**		
		**Grade I (11)**	**Grade II (48)**	**Grade III (31)**
(I)	Otsu	0.732 ± 0.151	0.659 ± 0.186	0.664 ± 0.174
(II)	UNet-LR	0.880 ± 0.127	0.862 ± 0.142	0.890 ± 0.099
(III)	Inc-PW	0.901 ± 0.072	0.882 ± 0.088	0.890 ± 0.095
(IV)	Mob-PW	0.887 ± 0.089	0.882 ± 0.092	0.890 ± 0.100
(V)	Mob-KM-PW	0.851 ± 0.111	0.872 ± 0.089	0.880 ± 0.088
(VI)	Mob-PW-UNet	0.936 ± 0.073	**0.931 ± 0.058**	**0.935 ± 0.083**
(VII)	Mob-PW-AGUNet	0.933 ± 0.082	0.926 ± 0.060	0.927 ± 0.083
(VIII)	Mob-PW-DAGUNet	0.935 ± 0.075	0.926 ± 0.058	0.929 ± 0.088
(IX)	Mob-PW-DoubleUNet	**0.942**±0.070	0.924 ± 0.066	0.934 ± 0.085

DSC, Dice Similarity Coefficient; Inc, InceptionV3; Mob, MobileNetV2; PW, Patch-wise; KM, *k*-means; LR, Low-resolution. Results are reported as mean ± standard deviation.

The best performing method, Mob-PW-UNet, took approximately ~58 s to run on a representative × 400 WSI using the CPU (see Table 3). Using the GPU, runtime was reduced to 40.26 s. The patch-wise method dominated the overall runtime, with ~1% of the total runtime being used on the refinement stage.

**Table 3:** Runtime measurements of the proposed method, Mob-PW-UNet.

	**Patch-wise**	**Refinement**	**Total**
OpenVINO	57.32 ± 0.20	0.75 ± 0.01	58.07 ± 0.20
TensorRT	39.88 ± 0.62	0.38 ± 0.00	40.26 ± 0.62

Experiments were repeated ten times and respective average and standard deviation are reported in seconds. OpenVINO and TensorRT were used as inference engines for CPU and GPU inference, respectively.

## 5. Discussion

In this paper, a novel approach for breast cancer segmentation in WSIs was proposed. The method, called H2G-Net, includes a cascaded CNN architecture combining high-resolution and global information with negligible increase in runtime. A random sampling scheme was also proposed, which extracts patches directly from the raw WSI format, handling multiple data imbalance challenges simultaneously. We made the pipeline and trained models openly available in FastPathology (27), enabling the use of the method without programming. To conduct our experiments, we developed a novel data set comprising 624 breast cancer WSIs annotated by pathologists. We have presented each component in the pipeline and assessed the impact of each component in an ablation study. Using multiple guiding components, we significantly improved segmentation performance, while reducing disk storage requirements compared to conventional training pipelines.

The best performing architectures utilized both low and high-resolution information from the WSI. A similar approach is used by pathologists when separating the tumor from surrounding tissue. The low-resolution image provides a coarse outline of the tumor, whereas higher resolution is often necessary for accurate delineation.

### 5.1. Cascaded design and related work

**Figure 4:**
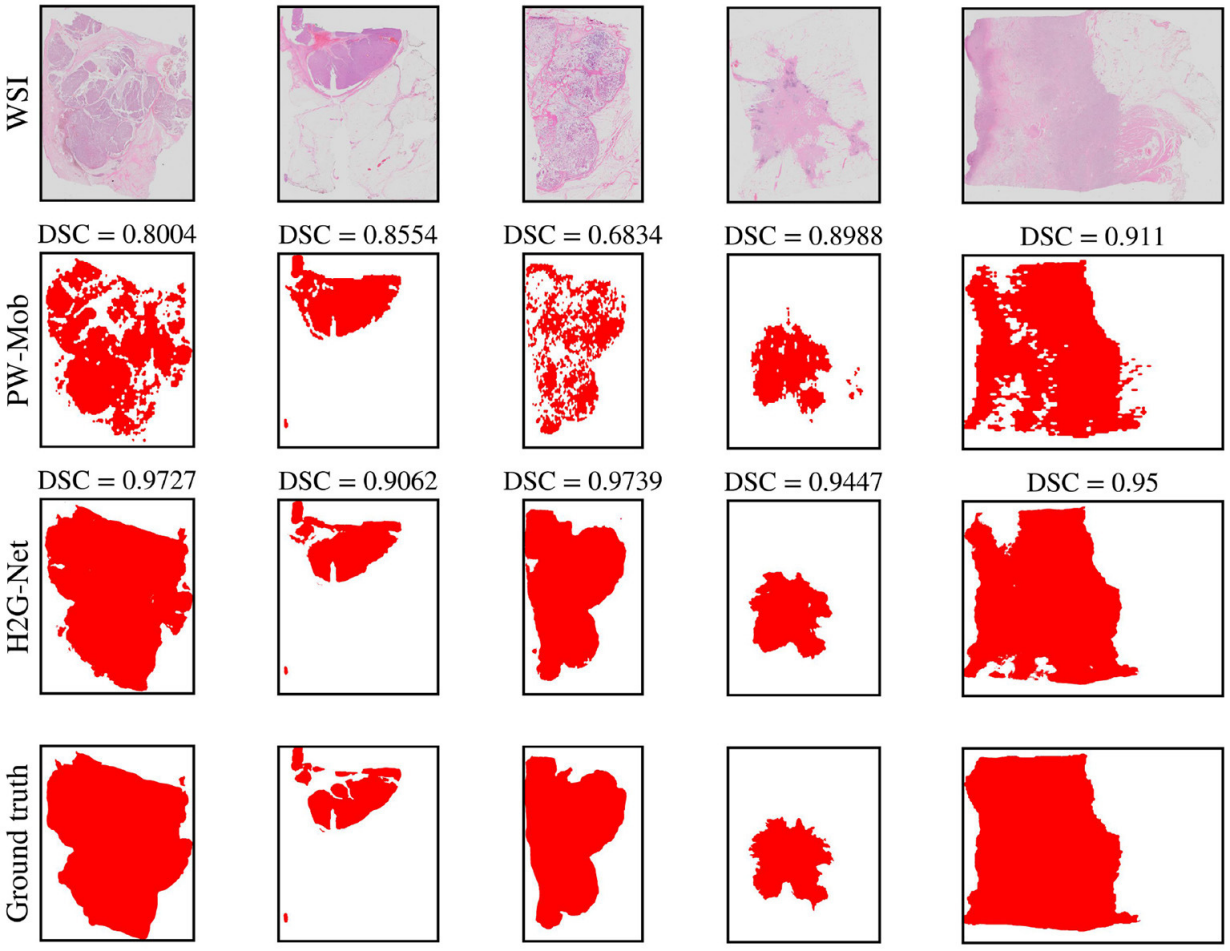
Qualitative segmentation results of five test set whole slide images (WSIs) comparing predictions of our method, H2G-Net, against a baseline method, PW-Mob, and the ground truth (pathologists' annotations). DSC, Dice Similarity Coefficient; PW, Patch-wise; Mob, MobileNetV2.

For segmentation, using low-resolution as the first step could reduce total runtime by filtering patches during preprocessing. However, UNet-LR, a U-Net using only low-resolution information, results in low sensitivity and should therefore not be the first step for breast cancer segmentation. In this work, we used a patch-wise, high-resolution method as a first step to optimize detection. A U-Net could then be trained at a later stage to refine the produced heatmap, using both the heatmap and the low-resolution WSI as input. Using the heatmap generated from the patch-wise method alone, some areas of the tumor, such as areas with abundant stromal or fatty tissue, may not be recognized, thus resulting in a fragmented heatmap (see Figure 4). We show that the network benefits from having the low-resolution image, together with the heatmap. Using this approach, the segmentation become more similar to the ground truth (the pathologists' annotations).

Tang et al. (50) used a two-step procedure to perform instance segmentation of objects in the Cityscapes data set (51). These images have an initial resolution of 1024 × 2048 pixels, but the classes of interest can easily be distinguished at lower resolution. In contrast to our design, they first performed semantic segmentation on the low-resolution, before refining the initial segmentation using a patch-wise design. They also used overlapping predictions along the border of the initial segmentation. Their approach might be an interesting refinement method to further improve the segmentation performance of our resulting low-resolution segmentation.

A similar refinement approach to (50) was used by (52) for cervical intraepithelial neoplasia segmentation of H&E stained WSIs. However, introducing a new network for border refinement will make the overall runtime longer and introduce more complexity to the final pipeline. Isensee et al. (53) conducted a similar two-step procedure for medical volumetric data. They first used a CAE applied on the downsampled version of the full 3D volume (CT/MRI). They then applied a 3D patch-wise refinement model, using both the local volumetric data (CT/MRI) and predicted heatmap as input.

Another similar architecture design to ours, was proposed by (54) for WSI classification of breast cancer. They also used a patch-wise model in the first step, before feeding the resulting heatmap to a second CNN that performed WSI-level classification. In addition, they used skip connections to propagate learned features from the patch-wise CNN to the latter CNN. Thus, our method could be seen as an adaption to their design applied to image segmentation. This style of skip connection is similar to the DoubleU-Net approach by (26). In this study, we did not explore skipping features from the classifier to the refinement network. This could be explored in future work. Our design is also similar to the work of (55), where a similar two-stage, cascaded CNN design was deployed, but for image registration of WSIs.

### 5.2. State-of-the-art comparison

An alternative approach, commonly used in the literature for other segmentation tasks (3), is to perform segmentation on patch-level. We argue that this approach is too slow and memory intensive for routine usage on low-end devices. As tumor segmentation is often the first step in any automatic cancer assessment pipeline, the method must be rapid. Furthermore, the annotations we used for training marked the outline of the tumor, thus including components like stromal tissue, inflammatory cells, and blood vessels, in addition to invasive epithelial cells. Within the tumor area, the different patches will have a varying morphological appearance. Therefore, using a patch-wise classifier alone, some patches may not contain sufficient information to make a qualified decision. This is even more challenging for semantic segmentation. Therefore, comparing our design to a high-resolution patch-wise segmentation models was not explored. The natural alternative state-of-the-art method could be to apply a patch-wise CNN classifier, which was the best performing method used for tumor detection and classification in the BACH challenge (4). We therefore performed an ablation study to make our design more easily comparable to current state-of-the-art approaches.

Comparison to state-of-the-art methods is challenging, as the data others have used are often not publicly available, may consist of other organs and cancer types, or have been annotated in a different manner. The BACH challenge data set includes 100 image patches from only ten annotated WSIs of invasive carcinomas. Another public data set is the CAMELYON17 (56) challenge data set comprising 1,399 annotated lymph node metastasis sections, where the task and tissue type are not comparable to our data. Lastly, the PAIP 2019 (57) challenge data set includes annotations made for the whole tumor area, using annotations similar to ours. The best performing team in the PAIP 2019 challenge used a patch-wise CAE and reached a Jaccard index of 0.7890, which translates to a DSC of 0.8821. This performance is comparable to the single-resolution approaches we tested (see Table 1). However, the sections they used were from liver cancer tissue. The training data set included only 50 annotated WSIs, in contrast to our data set, including 624 WSIs. Hence, a fair comparison cannot be made.

Cruz-Roa et al. (58) used a local data set of ~500 annotated WSIs from breast cancer for training and validating a patch-wise CNN classifier for detecting invasive breast cancer. They also proposed a sampling scheme based on Quasi-Monte Carlo sampling similar to ours, however, a heatmap refinement method was not explored. On their local validation set they achieved a DSC of 0.670, and on an independent test set of 195 WSIs from the The Cancer Genome Atlas (TCGA), they achieved a DSC of 0.759. Le et al. (59) used a local data set of 109 annotated WSIs from breast cancer to train and validate a patch-wise CNN classifier. They also used the same TCGA data set as (58) for evaluation, but used a ResNet-34 CNN backbone and a patch aggregation refinement method based on a sliding window pooling technique. They reported a DSC of 0.820.

As our best performing method is integrated into an open software (FastPathology), it is possible for researchers to make comparisons of their methods to ours, by running our pipeline on their own data. However, it is challenging to compare machine learning methods if they have not been trained on the same data, as machine learning models are a consequence of the training data. A large, public benchmark data set for breast cancer segmentation is therefore needed.

### 5.3. Architecture depth, patch generation, and clustering

An interesting observation in this study is that using the deepest and most complex network for tumor segmentation is not necessarily better. From Table 1, we found no statistical significant difference between using InceptionV3 and MobileNetV2 for detection of breast cancer tissue. A similar trend could be seen from the refinement network. Choosing more complex CAEs did not significantly improve segmentation performance. This could be due to data that did not cover all possible variations. The quality of the heatmap provided from the detection stage varied in some cases, making it challenging for the refinement network to improve the initial segmentation.

Reading patches from the raw WSI format is time consuming. It is therefore common to preprocess data before training. In this study, we sampled patches directly from the raw WSI format during training. This idea was recently proposed by (60). We further extended on their idea to make it more generic. The approach by (60) cannot handle larger batch sizes, as the cost of batch generation is not scalable. Thus, we used accumulated gradients to speed up batch generation, while simultaneously reducing GPU memory usage. We further introduced the concept of hierarchical sampling, which added direct support for balancing on multiple categories and labels. This design also added support for performing cluster-balancing end-to-end during training. The clustering method can also easily be substituted.

No benefit from using cluster-guiding to detect breast cancer tissue was observed, comparing Mob-KM-PW and Mob-PW by qualitative visual inspection. This was also observed in a study by (13) on a similar task. We hypothesize that the core reason why cluster-guiding did not improve performance in our use case may be that the fundamental problem of breast region segmentation does not necessary lie in handling data imbalance between tissue types. Due to the low field-of-view in patch-wise designs, there will most likely be patches where it is not possible to assign the correct label without also including global context. That is probably why our refinement network was more successful.

### 5.4. Future perspectives

Qaiser et al. (13) demonstrated improved performance by using a more advanced clustering design. Thus, in future work, substituting the traditional ImageNet features + PCA + *k*-means clustering approach with a more suited clustering design should be explored. A natural next step could then be to provide the predicted cluster heatmap with the tumor heatmap, as it would provide different, representation-informative, high-resolution information to the refinement network.

Multiple instance learning (MIL) is a promising approach that tackles the challenge of weak supervision and noisy ground truth (61). Exchanging the MIL design with the single-instance CNN classifier is possible. In this framework, one could still perform clustering in preprocessing, and sample patches to the bag, as done in a recent study (37). However, an interesting approach proposed by (62) was to perform clustering directly within the MIL design on bag-level. Using attention, one could train a network, not only to solve a task, but to learn subcategory structures in the data, while simultaneously filtering redundant clusters and noisy patches. This was demonstrated by (63). However, they did not assess the impact of the clustering component. Future work should involve replacing the single-instance CNN with the MIL design, incorporating clustering in an end-to-end fashion, and properly assessing its impact.

### 5.5. Strengths and limitations

The main strengths of the study are that the models were trained on a large set of breast cancer WSIs. Tumor annotations were created in a (semi-)automatic manner, and manually corrected by pathologists. To ensure consistency, all annotations were assessed by a pathologist experienced in breast cancer pathology. Validation studies were conducted using an independent test set. The performance of the different designs was also evaluated for each histological grade separately. An ablation study was performed to assess the impact of each component in the multi-step pipeline. The proposed design was validated against baseline methods, and the best method was integrated in an open platform, FastPathology (27).

The main limitations are that the models were trained using sections that were H&E-stained in the same laboratory and scanned in a single scanner. We demonstrated that the model generalized well to the test set, however, we have not tested our model on WSIs from other institutions. It is possible to carry out data augmentation to make the models more invariant, but it is challenging to mimic different staining and scanning effects (64). Thus, in the future, data from different laboratories and scanners will be added for training the models. We did not perform stain normalization as it would have added an additional layer of uncertainty and dependency in the pipeline. Furthermore, it would be interesting to assess the extent of generalization capability of our models to cancers of other origins, such as lung or gastrointestinal cancer. Lastly, our hierarchically-balanced sampling scheme was only used on a single task and not included in the ablation study.

## 6. Conclusion

Through our hybrid guiding scheme, we demonstrated a significant improvement in segmentation of breast cancer tumors from gigapixel histopathology images. The model outperformed single-resolution approaches and introduced a simple, fast, and accurate way to refine segmentation heatmaps, without the need for overlapping inference or ensembling. We also presented a hierarchical sampling scheme, that enabled patches to be streamed from the raw WSI format concurrently during training. Furthermore, we demonstrated that tissue type balancing can be performed end-to-end, using a novel loss function. The hierarchical sampling scheme and the novel loss function were introduced to make training methods more scalable and to reduce storage requirements.

## Data availability statement

The data sets presented in this article are not readily available because the ethical approval does not allow for making the data set publicly available. Requests to access the data sets should be directed to Marit Valla, marit.valla@ntnu.no. The source code to reproduce the experiments is made available at https://github.com/andreped/H2G-Net. The best-performing method is integrated into an open software, FastPathology, which can be downloaded at https://github.com/AICAN-Research/FAST-Pathology.

## Ethics statement

The studies involving human participants were reviewed and approved by the Central Norway Regional Committee for Medical and Health Research Ethics (reference numbers 2018/2141 and 836/2009). Written informed consent for participation was not required for this study in accordance with the national legislation and the institutional requirements.

## Author contributions

AP: conceptualization, methodology, investigation, annotation, writing—original draft, review, and editing, software, and validation. ES: methodology, investigation, supervision, writing—original draft, review, and editing, and software. TR, VD, and HP: annotation, writing—review and editing, and pathology expertise. DB: methodology, supervision, and writing—original draft, review, and editing. T-AN: methodology, investigation, and writing—review and editing. IR: conceptualization, methodology, supervision, and writing—original draft, review, and editing. MV: conceptualization, methodology, investigation, annotation, supervision, data curation, and writing—original draft, review, and editing, resources, pathology expertise, and project administration. All authors reviewed and approved the final version of the manuscript.

## Funding

This work was supported by The Liaison Committee for Education, Research and Innovation in Central Norway [Grant Number 2018/42794]; The Joint Research Committee between St. Olavs Hospital and the Faculty of Medicine and Health Sciences, NTNU (FFU) [Grant Number 2019/38882]; The Cancer Foundation, St. Olavs Hospital, Trondheim University Hospital [Grant Number 13/2021]; and The Clinic of Laboratory Medicine, St. Olavs Hospital, Trondheim University Hospital [Grant Number 2020/14728-49].

## Conflict of interest

The authors declare that the research was conducted in the absence of any commercial or financial relationships that could be construed as a potential conflict of interest.

## Publisher's note

All claims expressed in this article are solely those of the authors and do not necessarily represent those of their affiliated organizations, or those of the publisher, the editors and the reviewers. Any product that may be evaluated in this article, or claim that may be made by its manufacturer, is not guaranteed or endorsed by the publisher.

## Footnotes

^1^ https://github.com/libvips/libvips

^2^ https://github.com/andreped/vsi2tif

^3^ https://github.com/AICAN-Research/FAST-Pathology

^4^ https://github.com/andreped/H2G-Net

^5^ https://github.com/Peter554/StainTools
