# Supplementary material for: H2G-Net: A multi-resolution refinement approach for segmentation of breast cancer region in gigapixel histopathological images
Source: Front Med (Lausanne). 2022 Sep 14;9:971873. doi: 10.3389/fmed.2022.971873 (PMC9515451; doi:10.3389/fmed.2022.971873)
Supplement: Supplementary file 1 [file Data_Sheet_1.pdf]

Supplementary Material

1 SUPPLEMENTARY DATA

This document contains an additional table related to the manuscript entitled ”H2G-Net: A multi-resolution refinement approach for segmentation of breast cancer region in gigapixel histopathological images”.

Table S1 contains the adjusted p-values from the pairwise Tukey’s range tests referenced in section **3.3**. Red colour indicates no significance (p-value ≥ 0.05), green colour indicates slight significance (p-value $\in [0.001, 0.05]$, and light green colour indicates strong significance (p-value < 0.001). Pairwise comparisons were conducted on the Dice similarity coefficient, between the deep learning-based designs described in the main paper.

2 SUPPLEMENTARY TABLES AND FIGURES

Table S1. Adjusted p-values from the multiple pairwise Tukey’s range tests conducted on the test set ($n = 90$). Each row/column element represent different methods, with names that correspond to the same methods as described in the main paper. LR: low-resolution, Inc: InceptionV3, Mob: MobileNetV2, KM: k-means, PW: patch-wise.

	**Inc-PW**	**Mob-PW**	**Mob-KM-PW**	**Mob-PW-UNet**	**Mob-PW-AGUNet**	**Mob-PW-DAGUNet**	**Mob-PW-DoubleUNet**
**UNet-LR**	0.9	0.9	0.9	<0.001	0.002	0.001	<0.001
**Inc-PW**	-	0.9	0.9	0.012	0.049	0.041	0.03
**Mob-PW**	-	-	0.9	0.008	0.035	0.029	0.021
**Mob-KM-PW**	-	-	-	<0.001	<0.001	<0.001	<0.001
**Mob-PW-UNet**	-	-	-	-	0.9	0.9	0.9
**Mob-PW-AGUNet**	-	-	-	-	-	0.9	0.9
**Mob-PW-DAGUNet**	-	-	-	-	-	-	0.9
